# French lyophilized plasma versus normal saline for post-traumatic coagulopathy prevention and correction: PREHO-PLYO protocol for a multicenter randomized controlled clinical trial

**DOI:** 10.1186/s13063-020-4049-1

**Published:** 2020-01-22

**Authors:** Daniel Jost, Sabine Lemoine, Frederic Lemoine, Vincent Lanoe, Olga Maurin, Clément Derkenne, Marilyn Franchin Frattini, Maëlle Delacote, Edouard Seguineau, Anne Godefroy, Nicolas Hervault, Ludovic Delhaye, Nicolas Pouliquen, Emilie Louis-Delauriere, Julie Trichereau, Florian Roquet, Marina Salomé, Catherine Verret, René Bihannic, Romain Jouffroy, Benoit Frattini, Vivien Hong Tuan Ha, Pascal Dang-Minh, Stéphane Travers, Michel Bignand, Christophe Martinaud, Eliane Garrabe, Sylvain Ausset, Bertrand Prunet, Anne Sailliol, Jean Pierre Tourtier, for the PREHO-PLYO Study Group

**Affiliations:** 1 Paris Fire Brigade Medical Emergency Department, 1 place Jules Renard, 75017 Paris, France; 2 Department of Education, Research and Innovation, Service de Santé des Armées, 1 Place Alphonse Laveran, 75230 Paris, France; 3 French Military Health Service, Val de Grâce military hospital, 1, Place Alphonse Laveran, 75230 Paris, France; 4 French army blood transfusion center, 1 Rue du Lieutenant Raoul Batany, 92140 Clamart, France; 5 Department of Anesthesiology and Intensive Care, Percy military teaching hospital, 101 avenue Henri Barbusse, BP 406, 92141 Clamart, Cedex France; 6 French Military Research Institute, 1 place Valérie Andre, BP 73, 91223 Brétigny sur Orge, France; 7 Department of Anaesthesiology and Intensive Care, Begin military teaching hospital, 94160 Saint-Mande, France

**Keywords:** Post-trauma coagulopathy, Lyophilized plasma transfusion, Hemorrhagic shock, Shock index, Prehospital emergency care, Advanced trauma life support

## Abstract

**Background:**

Post-trauma bleeding induces an acute deficiency in clotting factors, which promotes bleeding and hemorrhagic shock. However, early plasma administration may reduce the severity of trauma-induced coagulopathy (TIC). Unlike fresh frozen plasma, which requires specific hospital logistics, French lyophilized plasma (FLYP) is storable at room temperature and compatible with all blood types, supporting its use in prehospital emergency care. We aim to test the hypothesis that by attenuating TIC, FLYP administered by prehospital emergency physicians would benefit the severely injured civilian patient at risk for hemorrhagic shock.

**Methods/design:**

This multicenter randomized clinical trial will include adults severely injured and at risk for hemorrhagic shock, with a systolic blood pressure < 70 mmHg or a Shock Index > 1.1. Two parallel groups of 70 patients will receive either FLYP or normal saline in addition to usual treatment. The primary endpoint is the International Normalized Ratio (INR) at hospital admission. Secondary endpoints are transfusion requirement, length of stay in the intensive care unit, survival rate at day 30, usability and safety related to FLYP use, and other biological coagulation parameters.

**Conclusion:**

With this trial, we aim to confirm the efficacy of FLYP in TIC and its safety in civilian prehospital care. The study results will contribute to optimizing guidelines for treating hemorrhagic shock in civilian settings.

**Trial registration:**

ClinicalTrials.gov, NCT02736812. Registered on 13 April 2016. The trial protocol has been approved by the French ethics committee (CPP 3342) and the French Agency for the Safety of Medicines and Health Products (IDRCB 2015-A00866–43).

## Background

Acute hemorrhage represents the leading cause of preventable death after severe trauma [1–4]. Trauma-induced coagulopathy (TIC) occurs early after severe trauma, resulting from multiple and complex pathophysiological mechanisms [5]. It evolves in three phases. The first phase consists of the activation of multiple hemostatic pathways in association with tissue damage and hypoperfusion. The second phase arises from factors related to treatment during resuscitation. The third phase occurs post-resuscitation and leads to a prothrombotic state [6]. Related hemostatic biological abnormalities include decreased fibrinogen, platelets, and coagulation factors, resulting in increased prothrombin time (PT). A positive association between hemostasis abnormalities and the severity of tissue hypoperfusion has been observed, especially for PT [7, 8]. Three well-established factors correlate with mortality during traumatic shock: acidosis, hypothermia, and coagulopathy [9, 10].

Previous studies have shown that 25% of civilian trauma patients and one-third of military trauma patients will present with a TIC, which is associated with a high mortality rate [11, 12]. Borgman et al., in a retrospective cohort with war injuries, were the first to report a sharp decrease in death rate after traumatic shock when a 1:1 plasma/packed red blood cell (RBC) ratio was initiated within 4 h after trauma occurrence [13]. Many subsequent findings in both civil and military traumatology have confirmed the results of this landmark study [14]. Despite their retrospective approach, these studies have confirmed the positive impact of a high ratio on the prognosis of bleeding traumas while showing that this benefit exists only with the achievement of this high ratio early in the patient management process [15–18]. This time window is in accordance with the hypothesis that the administration of plasma attenuates TIC by providing coagulation factors and that this effect is all the more important because the treatment is administered early.

Fresh frozen plasma (FFP) use is challenging in the prehospital setting. To date, FFP requires either a thawing phase of 30 min and then transport to the scene or storage at 4 °C after thawing, with a high risk of wastage. French lyophilized plasma (FLYP; in French “*Plasma lyophilisé*” or PLYO), initially developed by the French Military Blood Institute, has shown effectiveness in the management of patients at risk for hemorrhagic shock in the military setting [19–21]. FLYP has practical advantages that include storage at room temperature, easy reconstitution in less than 6 min, and compatibility with all blood groups. These features suggest possibilities for FLYP use in the prehospital setting for the management of severe traumatic bleeding.

The main objective of this study is to assess the effectiveness of prehospital FLYP administration in the treatment of post-traumatic coagulopathy in severely injured patients at risk for hemorrhagic shock. The study will complement the results of previous randomized trials that assessed the effect of prehospital plasma transfusion on morbidity and mortality in civilian trauma patients [22–24].

## Methods/design

### Trial design

**Fig. 1:**
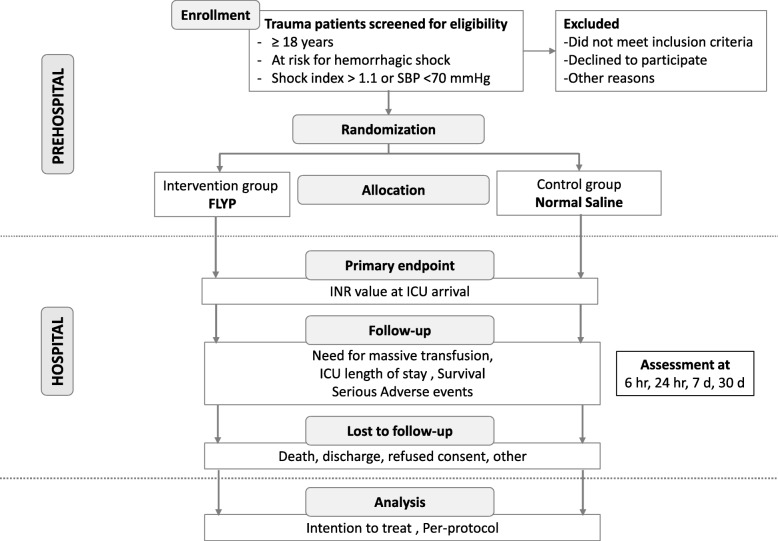
Study flow (CONSORT) for the PREHO PLYO trial. *SBP* systolic blood pressure, *FLYP* French lyophilised plasma, *ICU* intensive care unit

The Prehospital Lyophilized Plasma (PREHO-PLYO) trial is a multicenter randomized open-label controlled trial. Figure 1 presents the trial flow per the Consolidated Standards of Reporting Trials (i.e., CONSORT) guidelines [25, 26]. Additional file 1 presents the SPIRIT 2013 checklist (Recommended items to address in a clinical trial protocol and related documents).

### Setting and study population

In France, out-of-hospital emergency management is based on a two-tiered ambulance system. The first tier consists of basic life support (BLS) care provided mainly by firefighters. The second tier consists of physician-staffed mobile intensive care units (ICUs) that provide advanced life support (ALS) for life-threatening emergencies. Each medical team consists of an emergency physician, nurse, and driver. The emergency physicians will recruit severely injured patients at risk for hemorrhagic shock.

### Inclusion criteria

Inclusion criteria are as follows: prehospital patients who are severely injured and at risk for hemorrhagic shock and requiring an ALS team; age 18 years or more; and systolic blood pressure (SBP) < 70 mmHg or Shock Index (SI; calculated as heart rate (HR)/SBP) > 1.1.

### Non-inclusion criteria

Patients with any of the following will not be included: refusal to participate in the research; aged < 18 years; person deprived of liberty; pregnancy; known allergy to Amotosalen® and psoralen; previous prehospital administration of clotting factors other than Plyo; patient initially in cardiac arrest; or severe comorbid conditions with a not-to-be resuscitated status known since the prehospital setting.

### Interventions

All centers will perform the same planned experimental design regardless of the randomization arm Table 1. Patients randomly allocated to the plasma group will receive up to four units of FLYP with a dose of 15 to 20 ml.kg^− 1^. The control intervention is standard-of-care normal saline adapted to body weight at a dose of 15 to 20 ml.kg^− 1^. Patients will be blinded to the study treatment, but physicians will not. The infusion will be performed either by intravenous (IV) line or intra-bone line until the hemodynamic objective is reached, following the guidelines for post-traumatic hemorrhagic shock [27, 28].

**Table 1:** Template for the schedule of enrolment, interventions, and assessments per the SPIRIT 2013 Statement (Standard Protocol Items: Recommendations for Interventional Trials) [26]

	Prehospital	Hospital	
	ALS team arrival on scene	Admission at ICU	Follow-up
Eligibility screen	x		
Consent	x		x
Allocation to normal saline or FLYP	x		
Treatment Administration	x		
Patients Demographics	x		
Clinical/physiological Data	x	x	x
Injury Severity Score		x	
INR (point of care)	x	x	
INR (blood sample)	x	x	
Need for massive transfusion		x	
ICU length of stay			x
Death	x	x	x
Serious adverse Events	x	x	x

*ALS* advanced life support, *ICU* intensive care unit, *FLYP* French lyophilised plasma, *INR* international normalized ratio

### Randomization and treatment allocation

Randomization is a block size of 2 in a ratio of 1:1. The study statistician will secure the treatment allocation sequence codes throughout the trial. The French Army Blood Transfusion Center (Centre de Transfusion Sanguine des Armées (CTSA)) will prepare opaque bags in advance that will be identical in all points, except for treatment (either FLYP or normal saline) randomly assigned. These bags will be numbered from 1 to 140. The local French Blood Institute (Etablissement Français du Sang (EFS)) will store and dispense bags to each prehospital emergency medical service (see Appendix). Resuscitation ambulances will be equipped continuously with a two-bag set and ordered to open the smallest bag number after having validated the patient’s eligibility.

### Study outcomes

The primary outcome is the International Normalized Ratio (INR) at hospital admission. The secondary outcomes are as follows: need for massive transfusion or hospital-based administration of RBC, plasma, platelets, fibrinogen, and other coagulation factors; ICU length of stay; 30-day survival; FLYP prehospital usability in the civilian population (technical and logistical difficulties encountered with administration of FLYP); PT and fibrinogen at hospital admission; INR, fibrinogen, and PT differences between prehospital and hospital admission values; and FLYP prehospital safety (adverse event (AE) rate).

### Data collection

The main variables to be collected by the prehospital BLS and ALS teams are as follows: patient demographics and history; circumstances, mechanism, type, and site of the injury; clinical and biological status (SBP, diastolic blood pressure (DBP), mean blood pressure (MBP), HR, SI, respiratory rate, Glasgow Coma Scale score, body temperature, pulse oximetry, and hemoglobin and lactate levels when available); treatments and products administered (e.g., hemostatic device use (tourniquet, QuikClot®); volume expansion via IV or intra-bone infusion; sedation; use of vasopressor, tranexamic acid, or labile blood products; airway management (i.e., spontaneous breathing or mechanical ventilation)).

The main variables collected during hospitalization will be clinical and biological data, treatments administered, and patient outcomes. For all events, corresponding timeframes will be recorded, if available.

### Blood sampling

Blood samples will be collected by the prehospital team before treatment administration, using two 3.2% buffered sodium citrate Vacutainer® tubes. These will be stored at room temperature until arrival at the hospital, where the dosage will be evaluated. The filling level needed to allow laboratory analysis is 90% of tube capacity. To avoid confusion between pre- and in-hospital blood samples, a specific prehospital biological prescription sheet will identify each tube. Besides, if possible, INR and PT values will be determined for each patient from the prehospital phase, using a point-of-care coagulometer device (Coaguchek® Pro II System, Roche Diagnostics, Basel, Switzerland). The prehospital samples will be analyzed in the laboratory of the patient’s admission hospital. No biological collections will be conducted and no ancillary studies planned.

### Post-randomization treatment

Following the same measures as for study treatment administration, the physician will administer other indicated medications such as tranexamic acid, norepinephrine, and packed RBCs, if available. The ALS team will perform orotracheal intubation and chest drainage where indicated, in addition to all usual first aid procedures. Recommended blood pressure targets are SBP 90 mmHg without severe brain trauma and MBP 80 mmHg with severe brain trauma [29].

### Recruitment

**Fig. 2:**
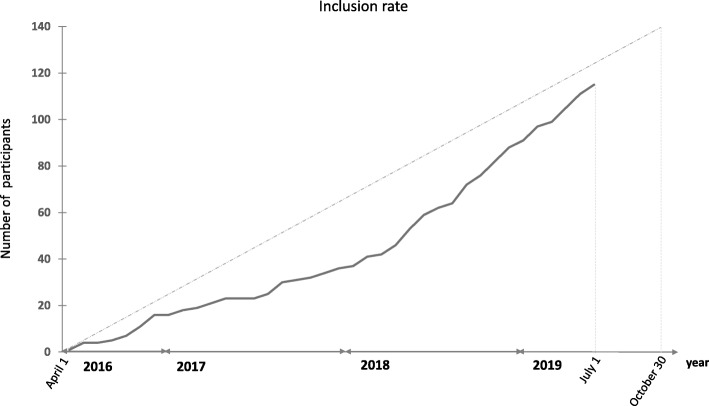
Inclusion rate up to the first 114 patients (1 July 2019). Allocation of the last of the 140 patients is anticipated to be in late October 2019. The *broken lines* represent the expected inclusion rate; the *solid line* represents included patients

Patient screening and inclusion started on 14 April 2016. As of July 1, 2019, 114 patients have been included. Recruitment is ongoing until the end of October 2019 (Fig. 2).

### Safety and adherence oversight

#### Data Safety Monitoring Board oversight

The Data Safety Monitoring Board (DSMB) will be composed of three independent physician experts in the field of transfusion and emergency medicine. They will be appointed to analyze AEs and severe AEs throughout the trial and will meet once every 6 months, but may meet at any time if necessary. No interim analysis is planned, but the DSMB will have access to all unblinded patient records to judge the quality of the study and the safety of the patients through a well-balanced risk–benefit ratio. This entity will have the right to stop the study if it appears that the benefit–risk ratio is unbalanced.

### Adverse events

Transfusion-associated circulatory overload (TACO) and transfusion acute lung injuries (TRALI) are the two main adverse transfusion-related events. The risk of TACO and TRALI is reduced inherently with FLYP production (less than ten different donors, plasma without HLA antibodies) [19]. In case of an adverse event, the FLYP transfusion will be immediately stopped and substituted by normal saline. The investigator will declare the main adverse event to the authorities within 24 h after its occurrence using a detailed written report.

### Protocol adherence

The prehospital emergency physicians and nurses involved in the trial will receive specific information about the study protocol as well as adapted training to administer the FLYP. All hospital laboratories will be informed about and able to analyze and distinguish prehospital and hospital blood samples.

### Data management

All data collected on the paper case report form will be entered into a database. A data manager will assess for validity, discrepancies, and quality assurance. All data with persistent discrepancies will be reported as missing values.

### Trial conduct auditing

The Project Management Group will meet monthly to review the trial conduct. The Trial Steering Group and the independent Data Monitoring and Ethics Committee will meet to review conduct throughout the trial period.

### Protocol amendments

The sponsor will notify the Ethics Committee of any change to the protocol via an online request. After the Ethics Committee has agreed, a copy of the revised protocol will be sent to the principal investigator to add to the Investigator Site File. The Head of Study Monitoring will verify that the revised protocol appears in the site file. Any deviations from the protocol will be fully documented using a breach report form. In the end, the protocol will be updated in the clinical trial registry.

### Statistics

The use of freeze-dried plasma has been assessed in severely traumatized patients with a before-and-after observational study in a military setting [20]. The “before” period consisted of an infusion of normal saline, and the “after” period involved FLYP transfusion. With a cohort of 120 patients, a significant difference between the two groups in terms of PT values was observed [20].

Based on these previous data, we calculated that a sample of 124 patients would provide 90% power to detect a mean difference between treatment groups of 0.3 in INR values at hospital admission, with a two-sided type I error of 0.05, assuming a standard deviation of 0.5. This sample size is corrected by a factor of 10% to account for missing informed consents and outcome data, for a final sample size of 70 participants per arm and 140 patients total.

The statistician, as well as the outcome assessor, will be blinded to patient randomization and group allocation. Analyses for the phase III trial primary outcome and secondary analyses are intention-to-treat. A per-protocol analysis will be carried out considering that some patients in the FLYP arm may not have received the treatment or may have received it only partially. Additionally, a priori subgroup analysis will be processed in a subpopulation of randomized patients with a confirmed diagnosis of hemorrhagic shock. No interim analysis is planned.

The quality of randomization will be assessed by ensuring that the distribution of characteristics in the two groups is balanced. Data will be summarized as medians with interquartile ranges for continuous variables and as counts with percentages for categorical variables. Comparisons between the groups for proportions will be assessed with Fisher’s exact test. The Mann–Whitney U test will be used to compare continuous variables.

Missing data will be presented comprehensively and detailed in a table. After an assessment of whether the assumptions underlying the validity of the multiple imputation are plausible, these values will be handled with multiple imputation.

For the primary outcome analysis, INR at hospital admission will be considered to have been performed at a comparable time in each patient and will be compared across the two arms with the Mann–Whitney U test. If the initial characteristics of patients are unbalanced between the two groups after randomization, an adjusted analysis will be considered with a regression method. Where appropriate, both adjusted and unadjusted results will be reported.

The transfusion requirement will be compared by the number of blood products and plasma derivatives transfused after arrival at the hospital, including packed RBCs, platelet concentrates, fibrinogen, coagulation factors, and FFP. The transfusion requirement will be assessed at 6 and 24 h, taking center into account as a stratifying variable.

For each arm, actuarial survival will be visualized using the Kaplan–Meier estimator and compared with the log-rank test to estimate the effect of FLYP on survival. A multivariable Cox proportional hazard regression model may be fitted in cases of potential imbalance.

The feasibility of administering FLYP will be judged based on interruption or non-administration of the experimental treatment, depending on the ability to respect the procedure and the use of a labile blood product according to the rules of good practice.

The average differential (prehospital and after-hospital admission) of coagulation parameters between the two groups will be studied with multilevel logistic regression modeling that will be fitted using a generalized estimating equation method to take into account time and both patient and hospital clustering. Where appropriate, this regression might be adjusted for unbalanced variables.

The number of days in the ICU between the two groups will be studied overall and among surviving patients. Concerning the safety outcome, the AE incidence will be compared between the two groups.

All statistical analyses will be performed with R software version 3.3.3 for Windows (R Foundation for Statistical Computing, Vienna, Austria) and STATA® software version 14.0 for Windows (STATACORP LLC, College Station, Texas, USA).

## Discussion

The administration of plasma is among the recommendations for the management of post-trauma hemorrhagic shock [27, 28]. The benefit of its early administration from the prehospital phase has recently been demonstrated with the use of FFP [22]. In a French trauma center, FLYP led to a more rapid, pronounced, and extended increase in fibrinogen concentrations and coagulopathy improvement than FFP in the initial management of trauma patients [30].

FLYP has thus far been used mainly in armed conflicts by military medical and surgical units deployed in foreign theaters of operations. These teams must navigate the logistical constraints of the operating environment and the need to have, without delay, therapeutic plasma to treat bleeding casualties. In the civilian world, FLYP could be used by health institutions that have significant logistical difficulties. These difficulties can preclude their ability to ensure a cold chain of subzero temperatures or, in extreme emergencies, to meet the need for an immediate therapeutic plasma supply because of delays until FFP is thawed and available [19]. The PREHO PLYO study is essential to assess both the possible benefit of FLYP on coagulopathy and its usability in patients of variable age suffering from various previous pathologies and being treated with a drug that may interfere with coagulation, as compared to young and physically trained soldiers without co-morbidity. The injury mechanism also differs in that, among soldiers, trauma is most often open or penetrating, whereas, among civilians, it is more likely to be closed. Finally, among the military, the distribution of wounded areas is not the same as for civilians because of the wearing of anti-ballistic protection.

The PREHO PLYO inclusion criteria are closely related to those used by three US Department of Defense-funded prehospital plasma trials [24, 31, 32]. Rather than using fixed thresholds of HR and SBP, we applied SI, which is the ratio between these two parameters. This index is easy to calculate at the bedside and makes it possible to detect patients in the compensated phase of hemorrhagic shock. Setting the SI threshold at > 1.1 corresponds to a five-fold higher risk of massive transfusion compared to a patient with a normal SI value (< 0.9) [33]. Concerning the choice of the INR as the primary endpoint, its standardized status and the possibility of its dosage at bedside make it an incomplete but easily accessible and feasible coagulation assessment parameter in real-world practice.

### Potential limitations

This trial involves several potential limitations. The first is related to the absence of a blind procedure for the prehospital medical team because it is not feasible to use a FLYP placebo. The second limitation is related to the difficulty prehospital teams may have in carrying out biological sampling in a technically perfect manner in the context of extreme emergency, for patients requiring many technical procedures within a very tight timeframe. We partially pre-empt this problem by proposing that the teams perform micro-venous sampling that they can immediately analyze using an onboard biology device. The third limitation is related to the consent of individuals in emergencies. Even if the emergency condition allows for a deferred time to obtain consent, we expect some refusals, which will require that the patients concerned be excluded from the statistical analysis. Because only surviving patients will be able to decline, survival rates may be lower than in reality and should be interpreted with caution.

## Conclusion

The PREHO PLYO clinical trial is the first study to evaluate the use of FLYP in prehospital settings. Its realization represents a stimulating challenge for prehospital emergency care. Its results will impact internal transfusion procedures and potentially extend the application of FLYP transfusion to patients who need it without any further delay.

## Trial status

This protocol version 6 is dated May 27, 2019. The first inclusion took place on April 14, 2016. The trial is in the recruitment phase until the end of October 2019 (planned).

### Ethical considerations

The trial protocol has been approved by the French Ethics Committee “Paris Ile de France 3: CPP 3342” and the French Agency for the Safety of Medicines and Health Products (IDRCB 2015-A00866–43). We will perform the study according to standard guidelines for clinical trials, to the Declaration of Helsinki, International Conference on Harmonization, and WHO Good Clinical Practice standards, including certification by an external audit. All conscious patients receive oral and written information and give their written informed consent. When the patient cannot give consent before the study (unconscious patient or high emergency case), the prehospital emergency physician will provide the allocated treatment in the patient’s best interest, and the investigator will collect deferred consent. Participants or their legal representative will be asked if they agree to use of their data should they choose to withdraw from the trial. They will also be asked for permission for the research team to share relevant data with people from the universities taking part in the research. Patients or their legal representative will have the possibility to withdraw their consent at any time. In such cases, the patient’s data will be excluded from the statistical analysis. The informed consent form for patients or family is available from the corresponding author on request.

### Recruitment of inclusion centers

Central ethics approval has been confirmed from the National Ethics Committee «Comité de protection des Personnes» (approval number CPP3342) and the authorization of the Competent Authority (French National Agency for Medicines and Health Products- Agence Nationale de Sécurité du médicament –ANSM IDRCB 2015-A00866–43 /approval 151536B63/EC-2015.003). The Sponsor will submit the recruitment of a new inclusion center to the National Ethics Committee and the competent authority if required.

### Confidentiality

The trial protocol is approved by the French Data Protection Agency (Commission Nationale Informatique et Liberté (CNIL)).

### Access to data

JT and FR will have access to the final trial dataset and has no contractual agreements that would limit access.

### Supplementary information

**Additional file 1.** SPIRIT 2013 checklist: Recommended items to address in a clinical trial protocol and related documents.

## Data Availability

The findings of the clinical trial will be published in a peer-reviewed international medical journal. The datasets obtained and analyzed during the current study will be available from the corresponding author on reasonable request. Further information about the study can be accessed at clinicaltrials.gov/show/ NCT02736812.

## Appendix

### French prehospital participating teams

Paris Fire Brigade Medical Emergency Dept

Firefighter Medical Emergency Dept of Marseille

French SAMU departments:

SAMU 75 Lariboisière Hospital, Assistance Publique Hôpitaux de Paris, France

SAMU 75 Necker enfants malades Hospital, Assistance Publique Hôpitaux de Paris, France

SAMU 94 Henri Mondor Hospital, Assistance Publique Hôpitaux de Paris, FranceSAMU 69, Hospices Civils de Lyon, France

SAMU 92, Beaujon Hospital, Assistance Publique Hôpitaux de Paris, Clichy, France

SAMU 74, Centre Hospitalier Annecy Genevois, France

SAMU 13, Assistance Publique Hôpitaux de Marseille, France

SAMU 29, University Hospital Brest, France

SAMU 64, University Hospital Pau, France

### French intensive care units participating teams

La Pitié Salpêtrière University Hospital, Assistance Publique Hôpitaux de Paris, Beaujon University Hospital, Assistance Publique Hôpitaux de Paris, Clichy

European Georges Pompidou Hospital, Assistance Publique Hôpitaux de Paris

Percy Military Teaching Hospital, Clamart

Begin Military Teaching Hospital, Saint Mandé

Bicêtre University Hospital, Assistance Publique Hôpitaux de Paris, Kremlin-Bicêtre

Henri Mondor University Hospital, Assistance Publique Hôpitaux de Paris.

Marseille-North Hospital, Assistance Publique Hôpitaux de Marseille

Timone Hospital, Assistance Publique Hôpitaux de Marseille

Laveran Military Teaching Hospital, Marseille

Edouard Herriot Hospital University Hospital, Hospices Civils de Lyon

Lyon-south Hospital University Hospital, Hospices Civils de Lyon

Annecy Genevois University Hospital, Annecy

Clermont Tonnerre Military Teaching Hospital, Brest

Hospital Center, Pau.

## Abbreviations

AE: Adverse event
ALS: Advanced life support
BLS: Basic life support
CTSA: Centre de Transfusion Sanguine des Armées
EFS: Etablissement Français du Sang
FLYP: French Lyophilized Plasma
HR: Heart rate
INR: International Normalized Ratio
MBP: Mean blood pressure
PT: Prothrombin time
RBC: Red blood cell
SBP: Systolic blood pressure
TACO: Transfusion-associated circulatory overload
TIC: Trauma-induced coagulopathy
TRALI: Transfusion-related acute lung injury
